# The Prediction Score of Acute Kidney Injury in Patients with Severe COVID-19 Infection

**DOI:** 10.3390/jcm12134412

**Published:** 2023-06-30

**Authors:** Suthiya Anumas, Supoj Chueachinda, Pichaya Tantiyavarong, Pattharawin Pattharanitima

**Affiliations:** 1 Chulabhorn International College of Medicine, Thammasat University, Pathum Thani 12120, Thailand; 2 Division of Nephrology, Department of Internal Medicine, Faculty of Medicine, Thammasat University, Pathum Thani 12120, Thailand

**Keywords:** acute kidney injury, COVID-19, risk factors, prediction

## Abstract

Background: The incidence and risk factors for acute kidney injury in COVID-19 patients vary across studies, and predicting models for AKI are limited. This study aimed to identify the risk factors for AKI in severe COVID-19 infection and develop a predictive model for AKI. Method: Data were collected from patients admitted to the ICU at Thammasat University Hospital in Thailand with PCR-confirmed COVID-19 between 1 January 2021, and 30 June 2022. Results: Among the 215 severe-COVID-19-infected patients, 102 (47.4%) experienced AKI. Of these, 45 (44.1%), 29 (28.4%), and 28 (27.4%) patients were classified as AKI stage 1, 2, and 3, respectively. AKI was associated with 30-day mortality. Multivariate logistic regression analysis revealed that prior diuretic use (odds ratio [OR] 7.87, 95% confidence interval [CI] 1.98–31.3; *p* = 0.003), use of a mechanical ventilator (MV) (OR 5.34, 95%CI 1.76–16.18; *p* = 0.003), and an APACHE II score ≥ 12 (OR 1.14, 95%CI 1.05–1.24; *p* = 0.002) were independent risk factors for AKI. A predictive model for AKI demonstrated good performance (AUROC 0.814, 95%CI 0.757–0.870). Conclusions: Our study identified risk factors for AKI in severe COVID-19 infection, including prior diuretic use, an APACHE II score ≥ 12, and the use of a MV. The predictive tool exhibited good performance for predicting AKI.

## 1. Introduction

COVID-19, caused by a novel coronavirus, has rapidly spread worldwide since its emergence in Wuhan, China, in December 2019. This highly contagious disease presents a spectrum of respiratory symptoms, ranging from mild upper respiratory tract infection to severe acute respiratory distress syndrome (ARDS). However, what is concerning is the increasing incidence of acute kidney injury (AKI) observed in severe cases of COVID-19, indicating kidney damage as an additional complication. Extensive research has shed light on the prevalence of AKI among patients with severe COVID-19 infection. The occurrence of AKI can vary significantly across different regions, with rates ranging from 10% to 70% [1,2,3,4,5] in individuals with COVID-19 pneumonia and surpassing 32% among those requiring intensive care unit admission [6]. AKI has emerged as a common and worrisome complication, increasing mortality associated with severe cases of COVID-19 [5].

Several studies have extensively investigated the risk factors associated with the development of AKI in COVID-19 patients. These investigations have identified various notable factors, including gender, advanced age, and the presence of underlying non-communicable diseases such as diabetes, hypertension, heart failure, cardiovascular disease, chronic kidney disease (CKD), chronic obstructive pulmonary disease, and peripheral vascular disease [5,7,8,9]. Furthermore, the severity of the COVID-19 infection itself has emerged as a critical determinant of AKI risk. It is crucial to acknowledge that different levels of COVID-19 severity may exhibit distinct risk factors linked to the development of AKI. This suggests that individuals with severe COVID-19 infection may face different risk factors compared to those with milder cases.

However, despite these significant findings, there is currently no established tool or method available to accurately predict the likelihood of developing AKI specifically in severe COVID-19 cases. Ongoing research and further investigation are essential to better understand and identify the precise risk factors associated with AKI in severe COVID-19 infection, and to develop effective prediction models. Such advancements in prognostic tools could greatly enhance clinical management and patient outcomes by enabling timely interventions and appropriate allocation of resources.

Therefore, the primary objective of this study is to investigate the risk factors associated with AKI in patients with severe COVID-19 infection. Through a comprehensive analysis of these factors, our aim is to develop an accurate prognostic apparatus that can assess the likelihood of AKI in such patients.

## 2. Materials and Methods

### 2.1. Study Design and Participants

This was a retrospective cohort study conducted between 1 January 2021 and 30 June 2022 at Thammasat University Hospital, Pathumthani Province, Thailand. Data were extracted from electronic medical records.

The inclusion criteria for this study consisted of COVID-19 patients who were at least 18 years old and had a confirmed diagnosis using the reverse transcription polymerase chain reaction (RT-PCR) method. The confirmation was completed by collecting secretions from the nasal cavity (nasopharyngeal swab) or through tracheal suction in cases where the patient was intubated. The patients were required to exhibit signs and symptoms that met the criteria for severe COVID-19 infection as defined by the WHO COVID-19 clinical management guidelines from 2023 [10], which were defined as oxygen saturation < 90% on room air, had signs of pneumonia, or had signs of severe respiratory distress. Additionally, the patients had to be admitted to the intensive care unit (ICU) at Thammasat University Hospital.

On the other hand, exclusion criteria were applied to patients who had end-stage kidney disease and were undergoing renal replacement therapy. Patients who could not provide complete information through the medical records were also excluded from the study.

Clinical data consisted of demographic characteristics, co-morbidities, current medication, clinical characteristics, laboratory results, and in-hospital treatment. The primary aim of the study was to ascertain the risk factors associated with AKI in individuals with severe COVID-19 infection. The secondary objectives involved assessing the rates of mortality and non-recovery from AKI among patients with severe COVID-19 infection and developing a prediction score to anticipate the occurrence of AKI in individuals with severe COVID-19 infection.

The AKI was defined by an absolute increase in serum creatinine levels of 0.3 mg/dL within 48 h of admission or an increase in serum creatinine levels greater than 1.5 times the baseline value within 7 days. According to the Kidney Disease Improving Global Outcome 2012 classification [11], the severity of acute kidney injury (AKI) is classified into three stages: stage 1 (serum creatinine increase to 1.5–1.9 times baseline or an increase of ≥0.3 mg/dL within 48 h), stage 2 (serum creatinine increase to 2.0–2.9 times baseline), or stage 3 (serum creatinine increase to 3.0 times baseline or more, or serum creatinine level ≥4.0 mg/dL, or initiation of renal replacement therapy). Additionally, we collected data on patients who required the initiation of kidney replacement therapy (KRT) during their hospitalization within the first 7 days. A patient was considered to have not recovered from AKI if their serum creatinine at discharge was more than 1.5 times the baseline creatinine level.

### 2.2. Statistical Analysis

Categorical variables were presented as frequencies and percentages, while numerical data were reported as means with standard deviations (SD) or medians with interquartile ranges (IQR), as appropriate. To compare the categorical and continuous data between the two groups, Fisher’s exact test and Wilcoxon’s rank sum test were employed, respectively.

To identify the factors associated with AKI in severe COVID-19 infection, both univariate and multivariate linear regression analyses were performed. Variables that showed a *p*-value less than 0.1 in the univariate logistic regression analysis were included in the multivariate logistic regression analysis. The strength of the association between predictors and the outcome was expressed as odds ratios [1] along with their corresponding 95% confidence intervals [12]. Statistical significance was set at a *p*-value of less than 0.05. Survival analysis was conducted using the Kaplan–Meier method, and the log-rank test was employed to assess differences in survival rates. Version 17.0/BE of STATA was used to conduct all statistical analyses.

The predictors from the multivariate logistic regression analysis were included in the model development process. The internal validation was assessed using a bootstrapping procedure with an 800-bootstrap sample in order to quantify the optimism of the developed model. The model was then adjusted with a shrinkage factor to create a final model. The log odds from the final model were used to create a prediction score. The area under the receiver operating characteristics curve (AUROC) was calculated to determine the performance of the prediction scores.

## 3. Results

### 3.1. Baseline Characteristics

Of the 215 severe-COVID-19-infected patients included, 102 (47.4%) experienced AKI and 113 (52.56%) did not have AKI. In total, 45 (44.1%), 29 (28.4%), and 28 (27.4%) patients had AKI KDIGO stage 1, 2, and 3, respectively.

The group of patients who developed AKI exhibited a notably higher prevalence of pre-existing CKD and prior diuretic use compared to the group without AKI. Upon admission, the AKI group demonstrated significantly elevated proportions of high Acute Physiology and Chronic Health Evaluation II (APACHE II) scores, occurrence of ARDS, leucocytosis, metabolic acidosis, hypoalbuminemia, high levels of serum potassium, use of oxygen therapy, and inotrope/vasopressor drug in contrast to the non-AKI group. The remaining variables investigated in this study displayed similar proportions between the two groups (Table 1).

### 3.2. Renal Outcomes

Among the AKI group, 10 patients (accounting for 9.8% of the total) required renal replacement therapy (RRT). The indications for RRT varied among the patients, and some individuals had multiple indications. The primary reasons for initiating RRT included volume overload in 9 patients (90%), electrolyte imbalance in 6 patients (50%), uremic symptoms in 5 patients (40%), and metabolic acidosis in 3 patients (30%). Among the AKI survivors, 25 patients (75.8%) recovered from AKI at hospital discharge.

### 3.3. Mortality

**Figure 1:**
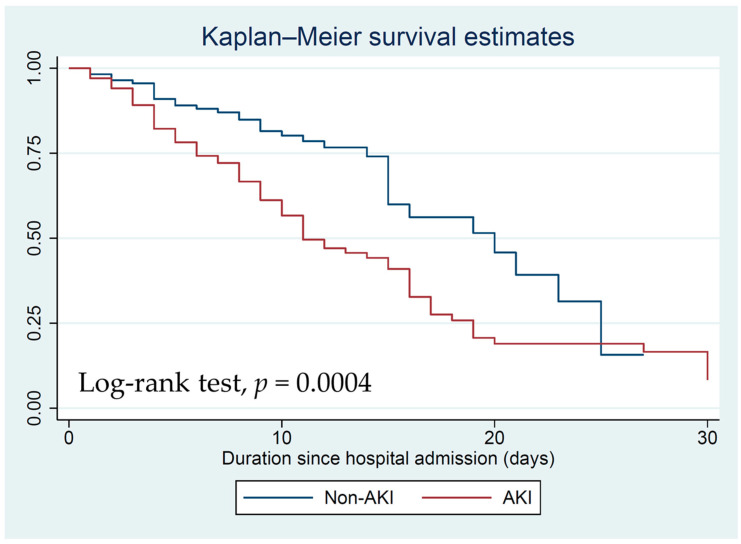
The Kaplan–Meier survival compares 30-day mortality between the AKI and the non-AKI groups.

**Figure 2:**
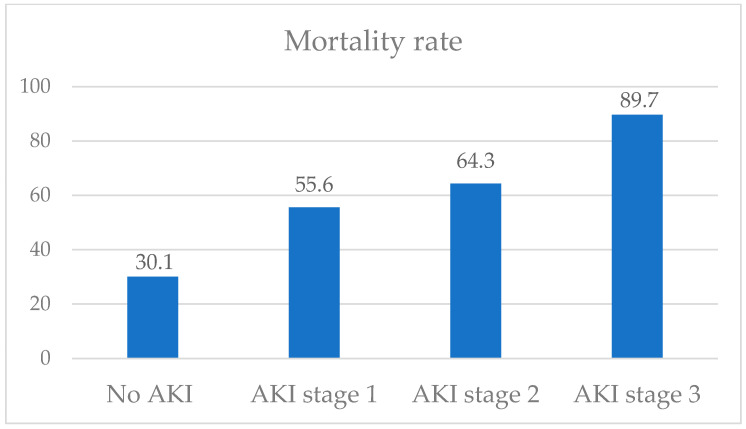
Mortality rate according to stage of AKI.

Upon discharge and within the 30-day period following the initial assessment, the AKI group exhibited a significantly higher association with 30-day mortality compared to the non-AKI group (Log-rank test, *p* = 0.0004) (Figure 1). In the AKI group, the mortality rate was 67.6% compared to 30.1% in the non-AKI group. Furthermore, within the AKI group, stage 3 AKI patients exhibited the highest mortality rate (89.7%) (Figure 2).

### 3.4. Risk Factors of AKI in Severe COVID-19 Infection

According to the results of univariate logistic regression analysis, several risk factors for AKI in COVID-19 patients were identified. These risk factors included age over 60 years, pre-existing CKD, prior diuretic use, APACHE II score of 12 or higher, serum albumin levels below 3.5 g/dL, leucocytosis, the need for inotrope/vasopressor drugs, and oxygen therapy.

Further analysis using multivariate logistic regression demonstrated that three variables independently associated with the development of AKI in COVID-19 patients were prior diuretic use (OR 8.21, 95% CI 2.03–33.28; *p* = 0.003), the need for mechanical ventilation (OR 5.09, 95% CI 1.73–14.96; *p* = 0.003), and an APACHE II score of 12 or higher (OR 2.96, 95% CI 1.42–6.19; *p* = 0.004) (Table 2).

### 3.5. Prediction Score

To develop a predictive score for determining AKI in severe COVID-19 patients, we included the significant and nearly significant risk factors with a *p*-value of 0.1 or less in our model. Logistic regression was employed to calculate the multiple correlation coefficients (Table 3). Furthermore, the goodness of fit of the model was assessed using the Hosmer–Lemeshow test, resulting in a *p*-value of 0.822 (95% CI 0.76–0.87).

To internally validate the model, bootstrapping was performed using an 800-bootstrap sample. The coefficients obtained from the developed model were adjusted for optimism by multiplying them with shrinkage factors of 0.93 (Table 3). The resulting optimism-adjusted linear equation was as follows: log odds (AKI in severe COVID patients) = −2.38 + 0.72 (Age > 60) + 1.93 (diuretic) + 1.16 (APACHE II score ≥ 12) + 0.70 (albumin < 3.5 g/dL) + 1.61 (mechanical ventilator). The optimism-adjusted model achieved an AUROC of 0.815 (95% CI 0.759–0.874), indicating its predictive performance.

**Figure 3:**
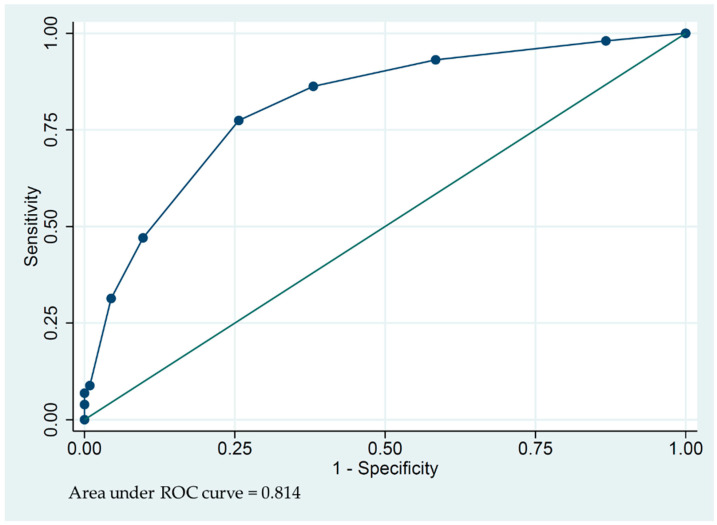
The receiver operating characteristic (ROC) [5] curves and AUROC of the final prediction model for AKI in severe COVID-19 patients.

According to the optimism-adjusted model, the denominator for the coefficients of the other predictors was set to the lowest value of 0.72. These coefficients were rounded to integers and utilized for score prediction. Weighting scores were assigned as follows: 1 point for individuals aged over 60 years, 3 points for those with prior diuretic use, 2 points for those with an APACHE II score of 12 or higher, 1 point for those with albumin < 3.5 g/dL, and 2 points for those with mechanical ventilator use (Table 4). The prediction score’s AUROC was found to be 0.814, with a 95% CI of 0.757 to 0.870. (Figure 3) A score of 3 or higher from the final model gave a sensitivity and specificity for predicting an AKI of 86.27% and 61.95%, respectively. In total, 67.18% of patients with a score of 3 or more developed AKI, while only 16.67% of patients with a score of less than 3 experienced AKI.

## 4. Discussion

This study demonstrated that 47.44% of patients with severe COVID-19 experienced AKI, which was also associated with 30-day mortality. The risk factors for AKI included prior diuretic use, an APACHE II score of 12 or higher, and the use of mechanical ventilation. A predictive model was developed and validated using these predictors, along with an age of over 60 years and serum albumin levels below 3.5 g/dL. The final model demonstrated good performance in predicting AKI in severe COVID-19 infection.

Previous studies have reported a wide range in the incidence of AKI in COVID-19 infection, varying between 10% and 70% [1,2,3,4,5]. A meta-analysis of these studies revealed a pooled incidence of approximately 19.45% [4]. This variation in incidence can be attributed to factors such as the severity of the infection. In our study, we specifically focused on severe COVID-19 infections, which might explain the slightly higher incidence of AKI observed. However, our study’s incidence was lower than that reported in studies conducted in Brazil [3] and the USA [13], which also focused on severe COVID-19 infections among patients admitted to the ICU. These studies reported the incidences of 71.2% and 56%, respectively. The differences in incidence rates could potentially be influenced by several factors, including differences in baseline characteristics and patient ethnicity, as suggested by a systematic review indicating that non-Asian populations tend to have a lower incidence of AKI compared to other populations [2].

The pathogenesis of AKI in COVID-19 infection involves various mechanisms. These include prerenal AKI resulting from dehydration and acute tubular necrosis (ATN) caused by factors such as prolonged prerenal AKI, medication, rhabdomyolysis, direct viral infection, cytokine storm, or lung–kidney crosstalk. Other mechanisms include glomerular injury, particularly associated with the APOL1 gene, acute interstitial nephritis due to medication, and hypercoagulable stages leading to microvascular injury [12,14,15]. Among these potential mechanisms, ATN was found to be the most common pathogenesis [16]. Considering these pathogenic processes, we included predictors that might be associated with each mechanism. Factors such as prior medication use, particularly diuretics, could contribute to prerenal AKI, ATN, or AIN. Blood pressure, APACHE II score, leukocytosis, and the use of mechanical ventilation were included as they represent the severity and inflammation of the disease which could be associated with ATN, cytokine storm, and/or lung–kidney crosstalk.

Our study identified predictors independently associated with severe AKI in COVID-19 infection, which included prior diuretic use, an APACHE II score of 12 or higher, and the use of mechanical ventilation. Compared to a meta-analysis conducted by Cai X et al. [8], which explored multiple risk factors associated with AKI in COVID-19 infection, we found some similarities. Older age, CKD, and mechanical ventilation were identified as common risk factors in their and our analyses.

Based on a meta-analysis, the pooled incidence of AKI in COVID-19 infection requiring KRT was reported to be 39.04% [4]. The incidence observed in our study was relatively lower; however, the indications for initiating KRT in our study were consistent with those reported in previous studies [17] which included hyperkalemia, oliguria, acidosis, high blood urea nitrogen (BUN), and pulmonary edema. Understanding the common indications for initiating KRT in AKI patients with COVID-19 infection can provide valuable insights for clinical management and guide appropriate interventions. In addition, Lumlertgul N et al. [17] also reported renal recovery rates of 81.6% and 90.09% for survivors at discharge and 90 days, respectively, which aligned well with our study.

According to a previous meta-analysis [4], AKI in COVID-19 has been associated with a poor prognosis and an increased mortality rate. This finding is consistent with the study conducted by Hsu CM et al. [13], which focused on critically ill patients. In the Hsu CM et al. study, the mortality rates progressively increased with each stage of AKI, with the highest mortality observed in AKI stage 3 and among patients requiring KRT. Significantly, our study’s findings were in line with the Hsu et al. study, as we also observed the highest mortality rate among patients with AKI stage 3. These findings reinforce the understanding that the severity of AKI, as indicated by its stage, is a critical factor in predicting patient outcomes and mortality in the context of COVID-19 infection. The identification of AKI stage 3 as a significant predictor of mortality emphasizes the importance of early detection and intervention for AKI in COVID-19 patients. Prompt recognition and appropriate management of AKI can potentially contribute to improved patient outcomes and reduced mortality rates.

Our predictor model considers both significant and nearly significant factors associated with acute kidney injury (AKI) in severe COVID-19 infection. We have identified several factors that increase the risk of AKI, including being over 60 years old, prior use of diuretics, an APACHE II score of 12 or higher, albumin levels below 3.5 g/dL, and the use of mechanical ventilation. The model’s AUROC value is 0.814, indicating its ability to predict AKI. Comparing our study to a previous study conducted by Palomba H. et al. [18], they also developed an AKI predictor model for COVID-19 patients admitted to the ICU. However, their predictor model included different factors such as diabetes, ACE inhibitor use, angiotensin receptor blocker (ARB) use, CKD, and hypertension. They did not clearly define the degree of disease severity in ICU patients, such as oxygen saturation. Additionally, the prevalence of AKI in their study was 33%, which is lower than in our study. This suggests that patients in our study may have had a higher severity of illness. Therefore, our predictor model, which includes factors associated with disease severity, not only preexisting conditions and/or medication use prior to ICU admission, but also the APACHE II score and mechanical ventilation use, has been found to be another significant predictor of AKI.

Our study developed a prediction score for assessing the risk of AKI specifically in severe COVID-19 infection, and it has demonstrated good performance. The factors included in our model are readily identifiable and commonly tested in ICU patients. This score can be effectively employed by clinicians to accurately predict the likelihood of AKI in this specific population. While some of these factors cannot be changed, their inclusion raises awareness among clinicians and enables early interventions for AKI prevention. However, it is important to acknowledge the limitations of our study. Firstly, as a single-center study, the generalizability of our findings to other settings may be limited. Secondly, we did not include urine output as a criterion for diagnosing AKI, which might have resulted in a lower incidence of AKI. Third, we did not consider the presence of patients under diuretic treatment during admission, which could be associated with poor clinical outcomes. Lastly, our study had a relatively small population. Therefore, further research with larger populations, multicenter collaborations, and comprehensive inclusion of all relevant criteria to identify AKI should be conducted to enhance the validity and applicability of our findings.

## 5. Conclusions

In severe COVID-19 patients, the prediction score comprised of age > 60, diuretic use, albumin 3.5 mg/dL, APACHE II score ≥ 12, and mechanical ventilation demonstrated a good performance in predicting AKI. In identifying patients at risk for AKI in severe COVID-19 cases, the predictive tool that incorporated these factors performed favorably. These findings could aid in the early intervention and management of AKI, thereby enhancing the prognosis for patients in this population.

## Figures and Tables

**Table 1:** Baseline characteristics.

Variables	All (N = 215)	Non-AKI (N = 113)	AKI (N = 102)	*p*-Value
Systolic BP, mmHg (SD)	131.01 (22.86)	133.70 (2.093)	128.04 (24.59)	0.07
Diastolic BP, mmHg (SD)	79.19 (15.42)	76.97 (12.96)	75.32 (17.79)	0.43
Pulse, bpm (SD)	90.96 (18.99)	89.34 (17.36)	92.76 (20.60)	0.18
APACHE II, score (SD)	11.92 (5.64)	9.49 (4.72)	14.60 (5.36)	<0.001
ARDS (%)	31 (14.42)	5 (4.42)	26 (25.49)	<0.001
Laboratory admission				
Hematocrit, % (SD)	36.71 (8.00)	37.56 (7.84)	35.75 (8.09)	0.10
WBC, cells/mm^3^ (SD)	8770.12 (5540.64)	7697.88 (4381.90)	9957 (6406.35)	0.002
Lymphocyte count, cells/mm^3^ (SD)	1096.14 (720.73)	1135.84 (553.66)	1052.18 (870.05)	0.40
Albumin, g/dL (SD)	3.37 (0.57)	3.50 (0.52)	3.22 (0.60)	0.0004
Baseline Cr, mg/dL (SD)	0.92 (0.42)	0.87 (0.42)	0.98 (0.42)	0.07
Sodium, mmol/L (SD)	133.86 (10.12)	132.87 (12.76)	134.96 (5.86)	0.13
Potassium, mmol/L (SD)	4.02 (0.80)	3.88 (0.60)	4.17 (0.95)	0.007
Bicarbonate mmol/L (SD)	22.46 (4.23)	23.31 (3.70)	21.53 (4.59)	0.002
Oxygen therapy (%)				<0.001
Cannula	57 (26.51)	44 (38.94)	13 (12.75)	
Mask with bag	1 (0.47)	1 (0.88)	0	
High flow	79 (36.74)	46 (40.71)	33 (32.35)	
BiPAP/CPAP	5 (2.33)	2 (1.77)	3 (2.94)	
Mechanical ventilation	73 (33.95)	20 (17.70)	53 (51.96)	
Inotrope/Vasopressor (%)	69 (32.09)	19 (16.81)	50 (49.02)	<0.001

Abbreviations: BP, blood pressure; bpm, beat per minute; APACHE II, Acute Physiology and Chronic Health Evaluation II; ARDS, acute respiratory distress syndromes; WBC, white blood cell; Cr, creatinine; BiPAP, bilevel positive airway pressure; CPAP, continuous positive airway pressure.

**Table 2:** Univariate and multivariate logistic regression analyses of risk factors associated with the development of AKI.

Variables	Univariate OR (95% CI)	*p*-Value	Multivariate OR (95% CI)	*p*-Value
Female	0.93 (0.53–01.61)	0.78		
Age > 60 years	2.94 (1.47–5.85)	0.002	2.28 (0.90–5.75)	0.08
BMI > 30 Kg/m^2^	0.70 (0.33–1.45)	0.34		
Comorbidities				
HT	1.50 (0.82–2.73)	0.18		
DM	1.23 (0.71–2.13)	0.47		
CKD	2.03 (1.03–4.00)	0.04	1.26 (0.53–2.95)	0.60
AF	2.64 (0.79–8.84)	0.12		
CAD	1.13 (0.52–2.44)	0.76		
COPD/asthma	0.84 (0.95–1.99)	0.69		
Cancer	1.04 (0.59–1.81)	0.89		
Previous Medication				
ACEi/ARB	0.85 (0.47–1.52)	0.58		
Diuretic	6.82 (1.92–24.17)	0.003	8.21 (2.03–33.28)	0.003
NSAIDs	1.10 (0.15–7.96)	0.92		
APACHE II score ≥ 12	5.65 (3.14–10.18)	<0.001	2.96 (1.42–6.19)	0.004
WBC (10^3^ cell/mm^3^)	1.07 (1.02–1.13)	0.01	1.05 (0.98–1.12)	0.17
Lymphocyte count (10^3^ cell/mm^3^)	0.85 (0.58–1.24)	0.40		
Albumin < 3.5 g/dL	2.53 (1.44–4.45)	0.001	1.81 (0.90–3.64)	0.10
Inotrope/Vasopressor	4.75 (2.54–8.90)	<0.001	0.96 (0.33–2.82)	0.95
Mechanical ventilator	5.03 (2.70–9.34)	<0.001	5.09 (1.73–14.96)	0.003

Abbreviations: BMI, body mass index; HT, hypertension; DM, diabetes mellitus; CKD, chronic kidney disease; AF, atrial fibrillation; CAD, coronary artery disease; COPD, chronic obstructive pulmonary disease; ACEi, angiotensin converting enzyme inhibitor; ARB, angiotensin II receptor blocker; NSAIDs, nonsteroidal anti-inflammatory drug; APACHE II, Acute Physiology and Chronic Health Evaluation II; WBC, white blood cell.

**Table 3:** Multiple correlation coefficients of risk factors for AKI in severe COVID-19 patients.

Variables	Multivariate Coeff. (95% CI) ^a^	*p*-Value	Multivariate Coeff. (95% CI) ^b^	*p*-Value
Age > 60 years	0.72 (−0.15 to 1.60)	0.10	0.72 (−0.11 to 1.55)	0.10
Prior diuretic use	2.07 (0.68 to 3.48)	0.004	1.93 (0.62 to 3.24)	0.004
APACHE II score ≥ 12	1.22 (0.53 to 1.91)	0.001	1.16 (0.51 to 1.81)	<0.001
Albumin < 3.5 g/dL	0.70 (0.02 to 1.38)	0.04	0.70 (0.05 to 1.34)	0.03
Mechanical ventilator	1.63 (0.89 to 2.37)	<0.001	1.61 (0.90 to 2.31)	<0.001

^a^ Developed model; ^b^ optimism-adjusted model. Abbreviations: APACHE II, Acute Physiology and Chronic Health Evaluation II.

**Table 4:** Prediction score for AKI in severe COVID-19.

Variables	Point
Age > 60 years	1
Prior Diuretic use	3
APACHE II score ≥ 12	2
Albumin < 3.5 g/dL	1
Mechanical ventilator	2

Abbreviations: APACHE II, Acute Physiology and Chronic Health Evaluation II.

## Data Availability

Data are available from the corresponding author upon reasonable request.
